# The Story of the Insane from Year to Year

**Published:** 1896-01-04

**Authors:** 


### THE STORY OF THE INSANE FROM YEAR TO YEAR.

Eigeth Series.

### DEVON COUNTY ASYLUM.

There was during the year 1894 a slight decrease in the number of patients. On January 1st the number was 1,070, and on December 31st, 1,063. There were 219 admissions, 75 recoveries, 31 relieved, and 88 deaths. The recoveries stand at 36·58 per cent. of the admissions, and the deaths at 8·31 on the average number resident. Hereditary influence accounts for 56 of the year's admissions, domestic trouble for

21, religion for 3, and intemperance for 10. Of the deaths, 28 were from spinal and cerebral diseases, 2 from dysentery, 1 from enteric fever, and 15 from phthisis. The asylum is practically full, and enlargements and alterations are in progress. The asylum is to be lighted with electricity at an expense of £6,000. The Committee of Visitors have followed the example set for them by Cornwall, Northampton, and Derby, and have formulated a pension scheme for their officers, attendants, and nurses. These are to receive as pension not more than one-fortieth nor less than one-fiftieth of the value of salary and allowances for every year of service, the minimum service being fifteen years and the maximum allowance twenty-six fortieths. This seems a reasonable scheme on the whole, and we congratulate the visitors on having sanctioned it and the officials on having received it. In our opinion, there should have been an age limit for compulsory retirement, without which a pension scheme has decided drawbacks. During the year one of the nurses received a pension of £40. The weekly rate of maintenance is 8s. 3d.

### SUNDERLAND BOROUGH ASYLUM.

This asylum issues an interesting report, but no statistics are given beyond those incorporated in the superintendent's report. We learn that the asylum was opened on May 21st, and it contained 292 patients on October 25th, leaving only 52 vacant beds—a number so small that the asylum will soon need enlarging. Dr. Elkin says that the buildings are well suited for their purposes, and the site pleasant and healthy. The asylum is warmed and ventilated on the plenum system, and is lighted by electricity. The Committee of Visitors were exceptionally fortunate in securing the services of such an able and experienced man as Dr. Elkin as medical superintendent, and we hope he has passed unhurt through the trying period of opening and organising a new asylum. We hear that no pension is attached to the appointment. If our information be correct we can only say that it is a great mistake on the part of the Committee of Visitors, and they cannot expect to retain the services of an efficient man. It practically means that an officer must die in harness, and no occupation is so trying as attendance on the insane. The visitors may take our word for it that the sooner they allow the pensions clause in the Lunacy Acts to follow the course intended by the Government the better it will be for everyone at their asylum.

### RAINHILL ASYLUM, LANCASHIRE.

On January 1st, 1894, this asylum contained 1,797 patients, and on December 31st the number had risen to 1,822. There were 371 admissions, 119 recoveries, 19 relieved, and 202 deaths. These numbers yield a percentage of 32·7 recoveries calculated on the admissions, and a percentage of 11·16 deaths calculated on the average number resident. Of the deaths 81 were caused by cerebral and spinal diseases, and 48 by consumption. Of the year's admissions 128 were owing to drink and 46 to hereditary influences. High as are these numbers they are probably under the truth; but at any rate they show that nearly 50 per cent. of the insanity was due to avoidable causes. We notice that a pathologist is employed at this asylum at a salary of £225 per annum, presumably with the object of investigating the causes of insanity in those patients who die in the asylum, and so inaugurate an improved treatment for the disease. We do not know what success has been met with in this object, but we are sure the ratepayers' money would be far better spent by making the pathologist into a peripatetic lecturer on the evils of intemperance and the dangers of marriage where hereditary disease exists. This would prevent the insanity occurring at all, and prevention is always better than cure. In insanity it is a thousand times better. In 1894 Dr. Wiglesworth had a murderous assault committed on him by a lunatic. He narrowly escaped with his life, and the wound was so serious that he was absent from his duties for five months. We heartily join with his friends in their congratulations on his recovery. The average cost per head per week is a fraction over 9s.

